# Notes and Notices

**Published:** 1897-03

**Authors:** 


### NOTES AND NOTICES.

DR. GIVEN'S SANITARIUM, Stamford Hall, at Stamford, Conn., is a cottage home for Nervous and Mental Diseases, with a separate department for Narcotic and Alcoholic Habitués. It is only one hour from New York, beautifully located, and arranged for the comfort of patients.

HOMŒOPATHS TO RECEIVE RECOGNITION.—Governor Stephens left this morning for Fulton to look after the management of the asylums at that place. He has this week been considering the make-up of the boards of managers of the various asylums, educational and eleemosynary institutions of the State. The terms of three members of each of the boards expired on the 1st day of February, and in the course of a few days the Governor will have named their successors.

The most decided change that Governor Stephens will make in the selection of his boards will be the appointment of management in charge of the insane asylum at Nevada, which will be favorable to the school of Homœopathy, and place that institution in charge of homœopathic physicians. This class of physicians have made a fight for recognition at the advent of each administration. They have on file in the executive office the strongest of arguments in their behalf, supported and strengthened by letters from prominent, conservative old-school physicians throughout the State. They claim that their patrons pay one-third of the taxes in the cities, and at least one-fourth in the State, therefore justice demands that they have charge of at least one asylum. The allopathic physicians now have charge of all of the asylums, insane, deaf and dumb, blind, and the penitenitary, reform school for boys, industrial home for girls, and the State University; in fact, all public institutions of the State, and a large percentage of them have signified liberality and have joined with the new school in the hope that the Governor will distribute patronage according to representation. Governor Stephens is himself a homœopath, and so is most of his family connection; but aside from this, he deems it but just and proper and right that the Nevada asylum should be placed under homœopathic management. He has definitely decided upon this course and will announce his various boards in a few days.—*St. Louis Globe-Democrat*, February 4th, 1897.

A Matter of Preference.—When doctors once yield to the allurements of a fad, they are powerful slow in breaking away. So slow, that if the appendicitis fever endures much longer there will not be an appendix in America—except in the ash-barrel. They tell us now that it is better to have it removed on general principles. And the joke of it is that during all this period of blood and terror the homœopaths, it appears, have been quietly treating it medicinally, seldom operating and rarely losing a case. But the Old School tells us the homœopath is a fool; and the homœopath, naturally, has his opinion of the Old School and its methods.

Well, as to choosing between the two fools, we have a leaning personally to pellets and appendix, in preference to the carving-knife and the ash-barrel.—*Life.*

### FUN FOR DOCTORS.

Dr. Kurnit—(writing a prescription) "Take this every morning."

Pat—"Divil a bit will I. Do yez t'ink oi'm a dumbed billy goat that yez kin fade me on a bit of paper?"

Jaymore—"Soakley was bitten by a mad dog last week."

Clogson—"Did he take hydrophobia as a result?"

Jaymore—"No, but the mad dog took delirium tremens."—*Washington Times.*

